# Ambient sulfur dioxide could have an impact on testicular volume from a observational study on a population of infertile male

**DOI:** 10.1186/s12894-020-00710-6

**Published:** 2020-10-02

**Authors:** Yu-An Chen, Yi-Kai Chang, Yann-Rong Su, Hong-Chiang Chang

**Affiliations:** 1 Department of Urology, National Taiwan University Hospital and National Taiwan University College of Medicine, No.1, Changde St., Zhongzheng Dist, Taipei City, 10048 Taiwan; 2 Department of Urology, Cardinal Tien Hospital, School of Medicine Fu-Jen Catholic University, No.362, Zhongzheng Rd., Xindian Dist, New Taipei City, 231 Taiwan; 3 Department of Urology, National Taiwan University Hospital Hsin-Chu Branch, NO.25, Lane 442, Sec.1, Jingguo Rd, Hsinchu City, 300 Taiwan

**Keywords:** Infertility, Air pollution, Sperm quality, Testicular volume

## Abstract

**Background:**

The effect of ambient pollutants on the male reproductive system is controversial. This retrospective study investigated the effect of environmental pollutants on male reproductive health.

**Methods:**

Male patients with primary infertility (*n* = 282) were identified from a single center between January 2016 and December 2017. Patients were physically examined for the presence of varicocele and for the volume of both testicles. Semen quality was measured in terms of the total sperm count, sperm concentration, and the percentage of sperm cells with motility and normal morphology. Data were acquired on the concentration of ambient pollutants, namely particulate matters of diameter < 2.5 μm, sulfur dioxide (SO_2_), nitrogen oxides (NO_x_), and ozone (O_3_), measured on daily and hourly basis, from the Environmental Protection Administration Executive Yuan, Taiwan. Individual exposure to pollutants was estimated based on the reported residential address of each participant. Statistical analysis indicated the effect of each pollutant on the testicular volume, sex hormone profile, and semen parameters.

**Results:**

The mean ± standard deviation of age was 36.7 ± 7.3 years. The average sperm count and concentration were 41.9 million/mL and 34.1 million/mL, respectively. The mean levels of serum testosterone, follicle-stimulating hormone, and luteinizing hormone were 3.57 ± 1.68 ng/mL, 7.59 ± 6.3 IU/L, and 4.68 ± 3.49 IU/L, respectively. According to the multivariate linear regression model, NO_x_ exposure was a risk factor for decreased sperm concentration and motility (*p* = 0.043 and 0.032). Furthermore, SO_2_ exposure was negatively associated and testicular volume (*p* < 0.01).

**Conclusions:**

NO_2_ and SO_2_ exposure were negatively associated with the seminal parameter and decreased testicular volume, respectively, in a population of men with infertility. However, additional prospective studies are needed to ascertain the cause–effect relation of current results.

## Background

Following industrialization, increasing attention has been focused on the effects of environmental toxicants on the human reproductive system. Occupational exposure to heavy metals, including arsenic, lead, and mercury, disrupts male reproductive functions through altered sex hormone levels and decreased testicular volume. Furthermore, environmental and occupational exposure to synthetic organic compounds, such as organochlorides and organophosphate, reduce sperm quality. Methyl parathion, a known pesticide, is a type of organophosphate [1–3]. Ambient pollutants resulting from the increasing number of automobiles and factories include particulate matters with aerodynamic diameter < 2.5 μm (PM_2.5_), nitrogen oxides (NO_x_), and sulfur oxides. These ambient pollutants have been shown to not only impair human fertility but also interfere with male reproductive functions [4–10].

Several mechanisms have been proposed to explain how inhaled ambient particles disrupt reproductive function. First, ambient particles could induce oxidative stress (OS) in the gonad along with increased proinflammatory cytokine gene expression [8]. Second, ambient pollutants could interrupt germ cell maturation through altering the expression of the gene regulating differentiation or apoptosis in mice germ cells [11, 12].

Previous studies on the disruption of male reproductive function due to air pollution have almost always only focused on semen quality. The outcomes of other parameters of reproductive function, particularly testicular volume and the presence of varicocele, were not evaluated [3, 13, 14]. Furthermore, the effect of a particular gaseous molecule, for example, sulfur oxides or ozone (O_3_), is still inconclusive, and the results of several studies have been conflicting each another [6, 12, 15–17]. This fueled our interest in conducting a study to evaluate the relationship between ambient pollutants and male reproductive functions based on semen parameters, sex hormone profile, and physical examination, namely examining testicular size and the presence of varicocele.

## Methods

### Ethic approval

The present study followed all standards for ethics regarding the experimentation and research. The institutional review board of the National Taiwan University approved our study and waived the informed consent requirement because of the retrospective design of the present study (approval number: 20190311 RIND).

### Study design

We retrospectively analyzed data from a medical chart database. Patient data were recorded, including age at the time of diagnosis, body mass index (BMI), and lifestyle factors, such as smoking and the presence of diabetes. Furthermore, blood test for fasting blood sugar and sex hormone profile were recorded. At least one semen sample was obtained from each participant. If a patient had more than one semen sample, the first sample was used for the analysis. Air pollution data were acquired from the Environmental Protection Administration Executive Yuan, Taiwan. Outcomes were seminal parameter, sex hormone profile, and change in testicular volume.

### Participants

We analyzed data of men diagnosed with primary infertility obtained from a single center (National Taiwan University Hospital, Taipei, Taiwan) from January 2016 to December 2017. Primary infertility was defined as the inability to conceive under an unprotected sexual encounter in ≥12 months as defined by the World Health Organization (WHO) [18].

Participants with aberrant chromosomes, previous chemotherapy/radiotherapy due to malignancy, or bone marrow transplantation were excluded. Furthermore, patients who were previously diagnosed with obstructive azoospermia, such as the absence of vas deferens or had received vasectomies or orchiectomy, were excluded.

### Physical examination

Physical examination consisted of determining the presence and grading of varicocele and recording the volume of both testicles. The testicular volume was estimated using the Prader orchidometer (Bayer, Müllerstraße 178, Berlin, Germany). Genital malformation, if any, was recorded. Body weight, height, and smoking habits, if any, were recorded.

### Blood and hormonal analysis

Venous blood samples were collected at 9:00–11:00 in the morning. Hormonal assays were conducted in accordance with the instructions of our institute. The testosterone level was recorded in ng/mL, and luteinizing hormone (LH) and follicle-stimulating hormone (FSH) levels were recorded in IU/L.

### Semen analysis

Patients were asked to practice abstinence for 3–5 days before semen collection. Each man was provided a wide-mouth plastic container. The semen sample was collected through masturbation and was sent to the laboratory at our institute. Each sample was smeared, stained, and preserved. The following semen variables were used as outcomes: total sperm count (million), sperm concentration (million/mL), percentage of motile sperm cells, and the percentage of sperm cells with normal morphology.

### Ambient pollutant data and analysis

Data were acquired from the Environmental Protection Administration Executive Yuan, Taiwan (https://airtw.epa.gov.tw). The concentrations of air pollutants, namely sulfur dioxide (SO_2_), O_3_, NO_x_ [nitrogen monoxide (NO) and nitrogen dioxide (NO_2_)], and PM_2.5_, were automatically recorded at 78 monitoring stations throughout Taiwan. The data collected from these stations were recorded automatically, and the measures were previously validated manually by using linear regression to check concentrations. The concentration of each pollutant was continuously measured on daily and hourly bases. The monthly average of each pollutant was calculated if ≥20 daily average records were available for a month. In this study, individual exposure to air pollution was estimated on the basis of current residential address reported by the study participants and the mean concentrations of air pollutants in the study period (January 2016 to December 2017).

### Statistical analysis

Values are expressed as the mean ± standard deviation. Linear regression with a regression coefficient β and a 95% confidence interval was applied to indicate the effect of each unit of pollutant on the testicular volume and semen parameter. Smoking and medical comorbidities, including obesity and the varicocele status, were adjusted in the multivariate regression model. Furthermore, we examined the effect of ambient pollutants on sex hormone by using the multivariate linear regression model. Because serum sex hormone is associated with testicular size, an individual linear regression was performed for testicular volume after adjusting for sex hormone. We set the significance level at *p* < 0.05. All data analyses were performed using SPSS version 12 (SPSS Inc., Chicago, IL, USA).

## Results

The demographic characteristics of the included participants are summarized in Table 1a and 1b. In the study, 282 participants residing in Taiwan were included. The mean (±standard deviation) age, BMI, and the sizes of the right and left testes of all the participants were 36.7 ± 7.3 years, 24.6 ± 4.2 kg/m^2^, and 14.18 ± 2.7 and 14.30 ± 2.4 mL, respectively. In total, 64 participants (22%) had varicocele, and 212 participants (77%) were nonsmokers. The majority of the participants (*n* = 265, 93%) resided in northern Taiwan; 3, 3, and 1% of the patients were from central, southern, and other parts of Taiwan, respectively.

**Table 1:** a and b. Demographic characteristics of the study participants

Characteristics	Mean ± standard deviation		Range
Age	36.7 ± 7.3 years		19–69
BMI	24.6 ± 4.2 kg/m^2^		17.3–36
Right testis	14.18 ± 2.7 ml		6–20
Left testis	14.30 ± 2.4 ml		5–20
Characteristics	N, total = 282	%	
Cigarette			
Smoker	65	23	
Nonsmoker	217	77	
Varicocele			
Present	64	22	
Absent	218	78	
Residency			
Northern Taiwan	265	93	
Central Taiwan	7	3	
Southern Taiwan	7	3	
Other	3	1	

### Sperm parameters

The average sperm count was 41.9 ± 56.6 million, and the average sperm concentration was 34.1 ± 38.8 million/mL. The percentages of sperms with total motility and normal morphology were 30.5 ± 16% and 23.6 ± 9.2%, respectively (Table 2). The mean values of the total sperm count, concentration, and normal morphology were higher in these participants than the cut-off values according to WHO standards [19]. However, the percentage of the progressively motile spermatozoa was low.

**Table 2:** Distribution of sperm parameters and sex hormone profile

	Mean ± standard deviation	Range
Sperm quality parameters		
Sperm count	41.9 ± 56.6 mln	0.25–280
Sperm concentration	34.1 ± 38.8 mln/ml	0.1–420
Sperm motility	30.5% ± 16%	0–75
Normal form	23.6% ± 9.2%	0–80
Sex hormone profile		
FSH	7.59 ± 6.3 IU/L	1.2–56.28
LH	4.68 ± 3.49 IU/L	0.32–33.5
Testosterone	3.57 ± 1.68 ng/mL	0.98–12.8

*Abbreviations*: *FSH* Follicle Stimulating Hormone, *LH* Luteinizing hormone

### Sex hormone profile

The sex hormone profile of the study population is summarized in Table 2. The mean levels of serum testosterone, FSH, and LH were 3.57 ± 1.68 ng/mL, 7.59 ± 6.3 IU/L, and 4.68 ± 3.49 IU/L, respectively. The mean testosterone level was higher than the cutoff value of hypogonadism as defined by the American urological association [20].

### Air pollution levels

**Fig. 1:**
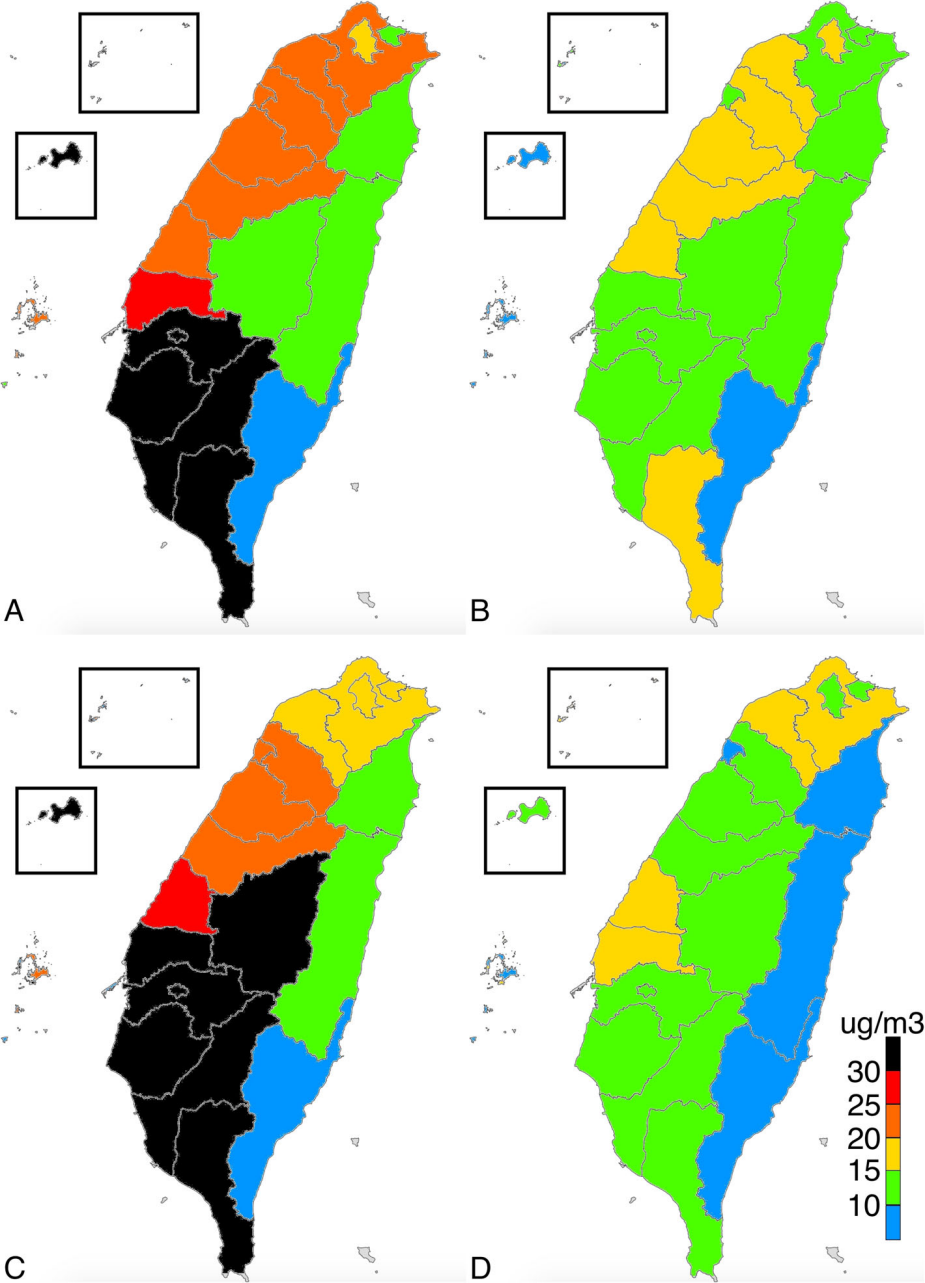
Distribution of 2.5-μm particulate matter in **a** January 2016; **b** July 2016; **c** January 2017; and **d** July 2017. (This work is adapted from the datasets of the National Land Surveying and Mapping Center MOI 2019–20, which is available to the public under the Open Government Data Licenses and is licensed under the Creative Commons Attribution-Share Alike 4.0 International license)

**Fig. 2:**
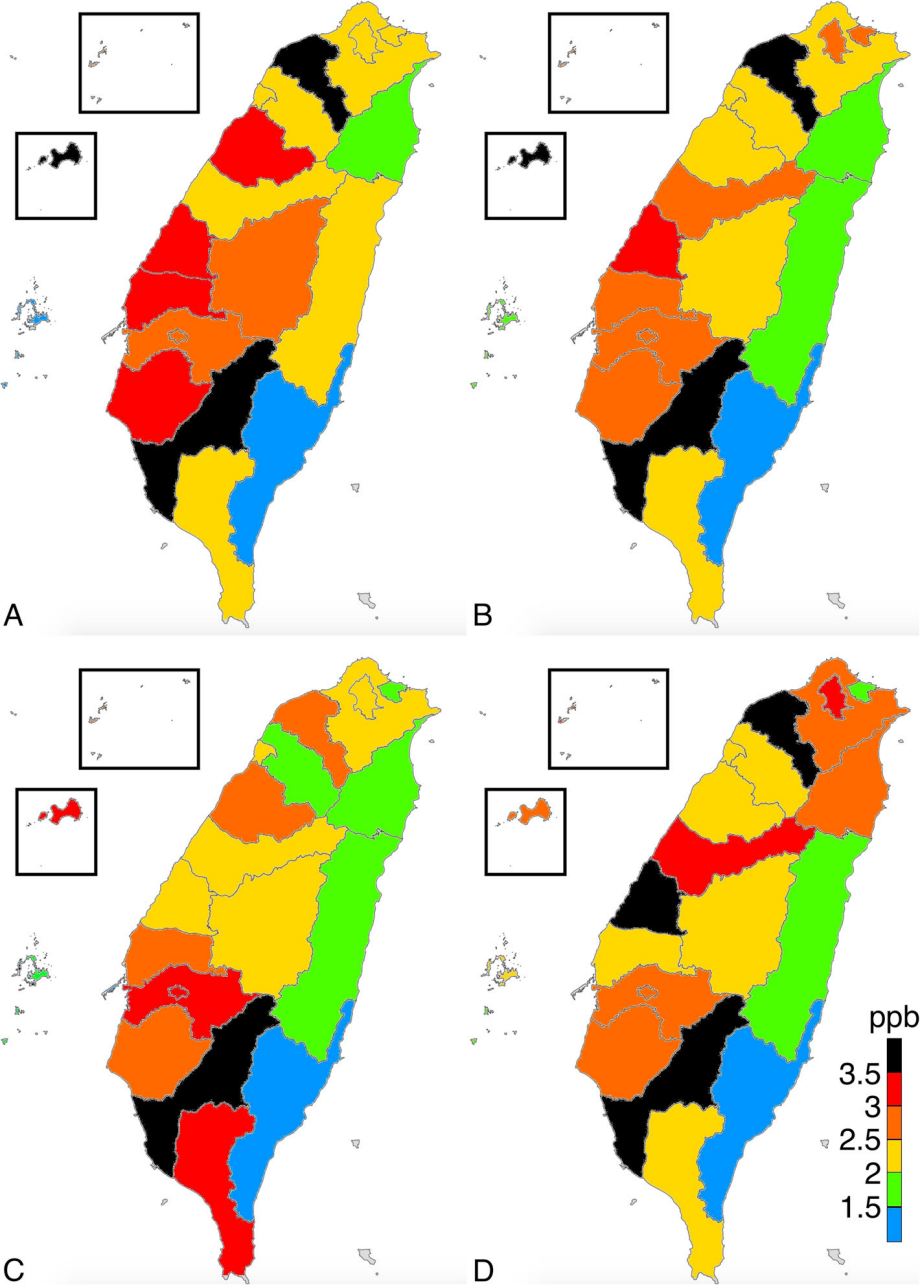
Distribution of sulfur dioxide in **a** January 2016; **b** July 2016; **c** January 2017; and **d** July 2017. (This work is adapted from the datasets of the National Land Surveying and Mapping Center MOI 2019–20, which is available to the public under the Open Government Data Licenses and is licensed under the Creative Commons Attribution-Share Alike 4.0 International license)

**Fig. 3:**
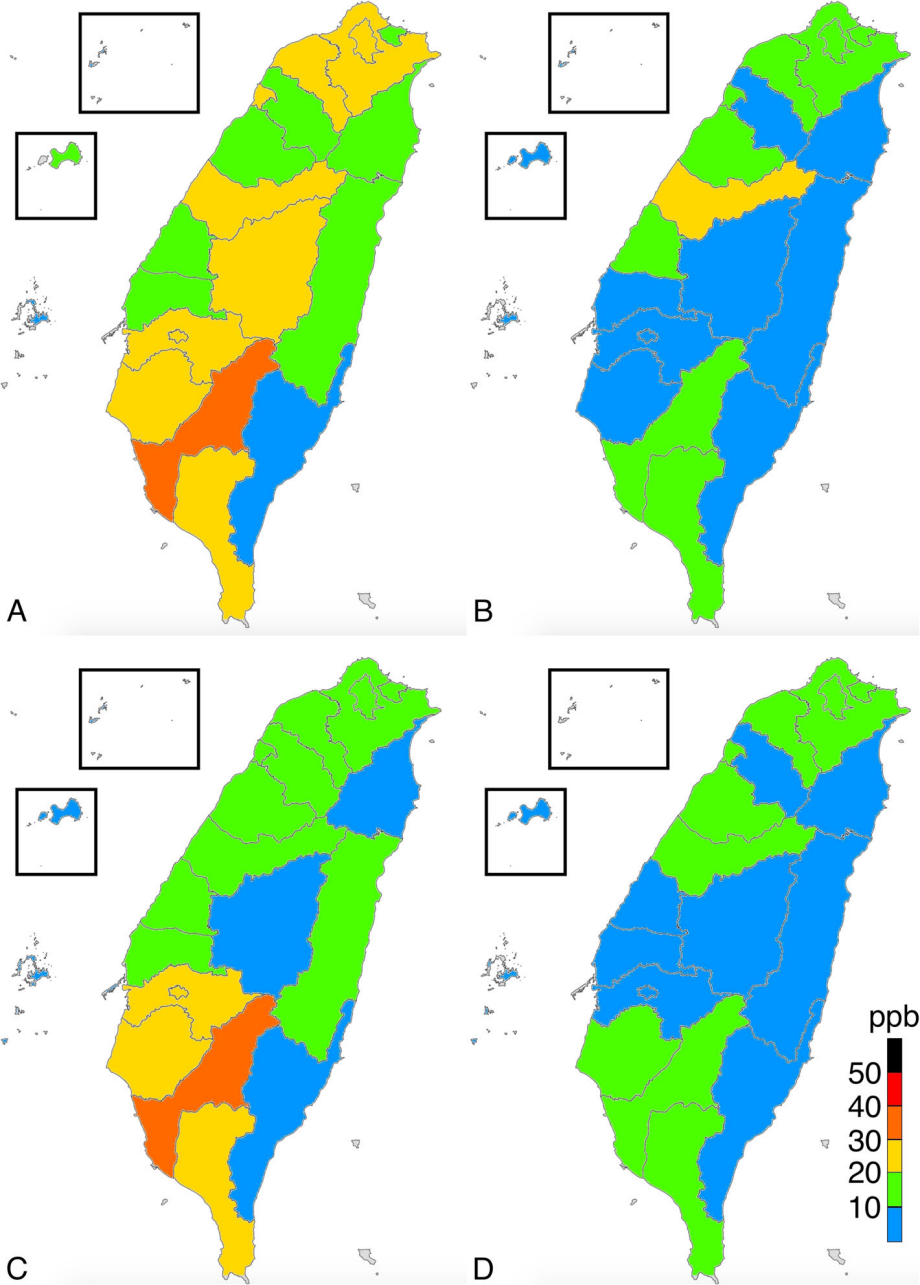
Distribution of nitrogen oxides in **a** January 2016; **b** July 2016; **c** January 2017; and **d** July 2017. (This work is adapted from the datasets of the National Land Surveying and Mapping Center MOI 2019–20, which is available to the public under the Open Government Data Licenses and is licensed under the Creative Commons Attribution-Share Alike 4.0 International license)

**Fig. 4:**
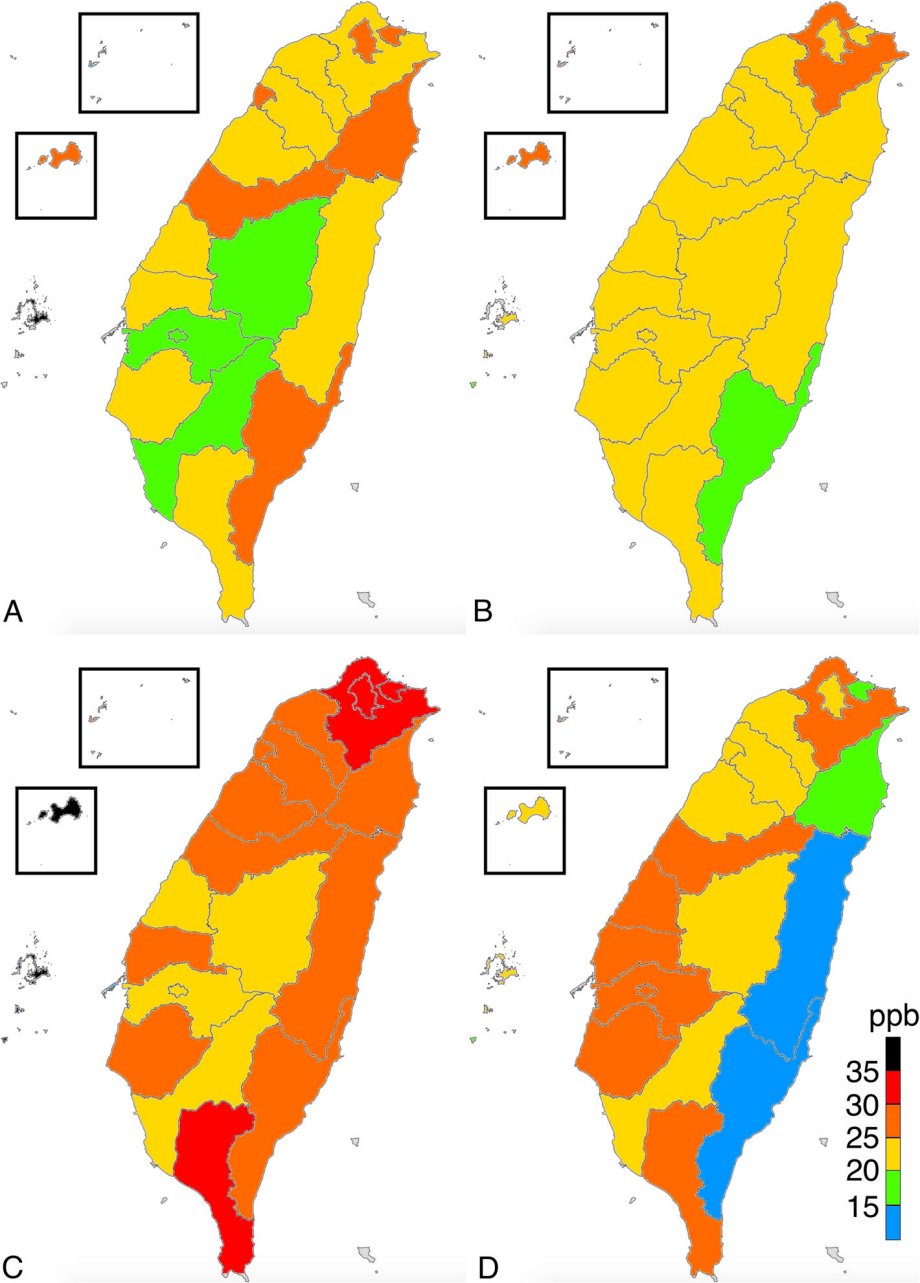
Distribution of ozone in **a** January 2016; **b** July 2016; **c** January 2017; and **d** July 2017. (This work is adapted from the datasets of the National Land Surveying and Mapping Center MOI 2019–20, which is available to the public under the Open Government Data Licenses and is licensed under the Creative Commons Attribution-Share Alike 4.0 International license)

**Fig. 5:**
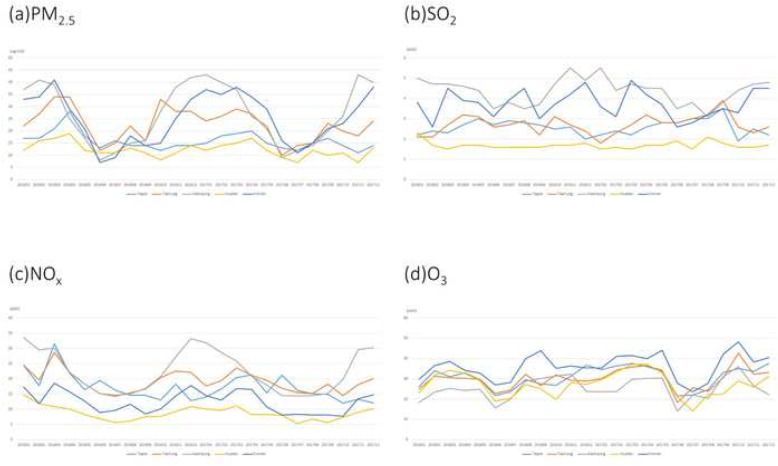
Ambient pollutants concentration in Taiwan from January 2016 to December 2017: **a** particulate matter of 2.5 μm; **b** sulfur dioxide; **c** nitrogen oxides; and **d** ozone

The average levels of PM_2.5_, O_3,_ SO_2_, and NO_x_ were 18.0 μg/m^3^ (range = 10.9–27.2 μg/m^3^), 27.3 ppb (parts per billion) (range = 21.0–40.6 ppb), 2.9 ppb (range = 1.7–5.6 ppb), and 29.0 (range = 3.7–91.9 ppb), respectively. According to WHO air quality guidelines, the recommended annual mean for PM_2.5_, O_3_, SO_2_, and NO_2_ were 10 μg/m^3^, 100 ppb, 20 ppb, and 40 ppb, respectively [21]. Except for PM_2.5_, the mean concentrations of particulate matter in Taiwan were within reference values suggested by the WHO air quality guidelines. The eastern part of Taiwan, which consists of mostly rural regions, had low SO_2_, NO_x_, and PM_2.5_ levels. The distribution of O_3_ did not considerably vary between the different parts of Taiwan, but a high O_3_ level was observed in Kinmen, an island located on the east coast of China. All ambient pollutants were at the highest concentration in the southern part of Taiwan, including Tainan, Kaohsiung, and Kinmen, in all seasons. The PM_2.5_ level was high in winter and low in summer, and the regional concentration difference of PM_2.5_ was particularly obvious in January and February (Figs. 1, 2, 3, 4, 5).

Tables 3, 4, 5 summarize multiple regression analysis results for the semen parameter, sex hormone profile, and testicular volume after adjusting for blood sugar, BMI, varicocele, and smoking. The exposed mean concentrations of NO_x_ in the study period were negatively associated with the sperm concentration and total motility (*p* < 0.05). No statistically significant relationship was observed between exposure to PM_2.5_, SO_2_ or O_3_ and sperm parameters. In this study, linear regression did not show an effect of ambient pollutant on the sex hormone profile. Only age was found to be an independent predicting factor for increased follicle stimulating hormone (Table 4).

**Table 3:** Multivariable linear regression by using a sperm parameter for exposure to ambient pollutants

Sperm parameters	Regression coefficients (95% confidence interval)	*p* value
Sperm count		
PM2.5	0.896 (−16.113 to 40.225)	0.382
SO_2_	−0.891 (− 169.427 to 68.260)	0.384
NOx	−1.136 (− 12.614 to 3.738)	0.270
O_3_	−0.196 (−18.947 to 15.698)	0.846
Sperm concentration		
PM2.5	0.109 (−13.821 to 15.340)	0.914
SO_2_	−1.302 (−99.796 to 23.235)	0.208
NOx	−2.174 (−8.627 to −0.164)	0.043*
O_3_	−1.749 (−16.458 to 1.475)	0.096
Sperm motility		
PM2.5	−0.303 (−7.508 to 5.608)	0.765
SO_2_	0.049 (−27.026 to 28.308)	0.962
NOx	−2.321 (−4.014 to −0.207)	0.032*
O_3_	−1.248 (−6.437 to 1.624)	0.227
Sperm normal form		
PM2.5	1.248 (−3.566 to 13.322)	0.234
SO_2_	−0.617 (−46.216 to 25.681)	0.548
NOx	1.400 (−1.147 to 5.369)	0.185
O_3_	0.992 (−4.546 to 12.267)	0.339

*p < 0.05

**Table 4:** Multivariable linear regression by using sex hormone for exposure to ambient pollutants

Sex hormone	Regression coefficients (95% confidence interval)	*p* value
FSH		
PM2.5	0.073 (−0.651 to 0.931)	0.704
SO_2_	0.262 (−2.613 to 7.24)	0.324
NOx	−0.431 (−0.218 to 0.004)	0.057
O_3_	0.122 (−0.503 to 0.852)	0.582
Age	1.053 (0.290 to 0.639)	< 0.01^**^
LH		
PM2.5	0.014 (−0.357 to 0.371)	0.966
SO_2_	0.170 (−1.857 to 2.680)	0.697
NOx	0.276 (−0.032 to 0.070)	0.436
O_3_	0.559 (−0.093 to 0.531)	0.150
Testosterone		
PM2.5	0.052 (−0.50 to 0.576)	0.880
SO_2_	−0.376 (−4.572 to 2.042)	0.421
NOx	0.156 (−0.061 to 0.090)	0.678
O_3_	−0.044 (− 0.469 to 0.424)	0.914

*Abbreviations*: *FSH* Follicle Stimulating Hormone, *LH* Luteinizing hormone, **p < 0.01

**Table 5:** Multivariable linear regression by using the testicular volume for exposure of ambient pollutant

	Regression coefficients (95% confidence interval)	*p* value
Right testicle		
PM2.5	−0.472 (− 0.956 to 0.607)	0.643
SO_2_	−3.101 (−8.279 to −1.575)	0.006**
NOx	−0.967 (− 0.376 to 0.140)	0.347
O_3_	−1.337 (− 1.007 to 0.226)	0.199
Left testicle		
PM2.5	0.472 (−0.956 to 0.607)	0.643
SO_2_	−3.101 (−8.279 to −1.575)	0.006**
NOx	−0.967 (− 0.376 to 0.140)	0.347
O_3_	−1.337 (− 1.007 to 0.226)	0.199

**p < 0.01

The testicular volume was negatively associated with the exposed mean concentration of SO_2_ (*p* < 0.01). The effect of SO_2_ on testicular volume was statistically significant even after adjusting for sex hormone (Table 6).

**Table 6:** Multivariable linear regression by using the testicular volume for exposure of ambient pollutant after adjusting for sex hormone

	Regression coefficients (95% confidence interval)	*p* value
Right testicle		
PM2.5	0.147 (−0.492 to 1.031)	0.476
SO_2_	−0.520 (−4.696 to − 0.0474)	0.018*
NOx	0.170 (−0.067 to 0.155)	0.424
O_3_	−0.188 (− 0.834 to 0.317)	0.366
Left testicle		
PM2.5	0.141 (−0.465 to 0.929)	0.502
SO_2_	−0.506 (−4.193 to − 0.328)	0.023*
NOx	0.199 (−0.055 to 0.147)	0.362
O_3_	−0.009 (− 0.538 to 0.516)	0.967

*p < 0.01

## Discussion

The current study investigated the effects of air pollutants on the reproductive health of men with infertility. As air pollutants are known to be hazardous to humans, it is unethical to conduct an interventional study on humans. Most studies on the effects of ambient pollutants on humans have been retrospective or observational, and the majority of them focused solely on the effects of pollutants on semen quality [6, 10, 13, 15–17, 22, 23].

Observational studies analyzing the effect of SO_2_ and NO_2_ on sperm quality have reported an unfavorable effect of these ambient particles on the sperm concentration and total count [9, 10, 15]. Our study suggested that NO_2_ exposure was negatively associated with the sperm concentration and sperm motility, which is consistent with the finding of Broggia [9]. Nevertheless, Zhang reported that SO_2,_ NO_2_, and PMs were not associated with poor semen quality [16].

The reported effect of O_3_ on the reproductive function of men was deleterious. Data obtained from country-wide air quality monitoring showed high O_3_ exposure to be an independent factor predicting low semen quality, including total sperm count, sperm concentration, and percentage of normal form [16, 22, 24]. However, our results did not show a significant association between O_3_ and seminal parameters. The inconsistency with the previous literature could be due to the small sample size and retrospective study design, which failed to detect statistically significant effects of O_3_ on male reproductive function.

The detailed mechanism through which ambient pollutants interfere with the male reproductive system is not well elucidated. One of the most frequently proposed mechanisms is the induction of OS in the reproductive system.

A rat model biopsy showed that PM_2.5_ increased OS in the testes. First, expression of superoxide dismutase (SOD) decreased, which is one of the crucial enzymes that protect cells from reactive oxygen species (ROS). Second, an increase in heme oxygenase (HO) was observed. HO participates in ROS metabolism. An increased HO level signified increased OS [25].

Similarly, a testicular biopsy of SO_2_-exposed mice showed decreased expression of antioxidation enzymes, including SOD and glutathione peroxidase (GPx). GPx catalyzed the reduction of peroxide radicals to alcohols and oxygen and thereby reduced OS [7].

NO_2_ and O_3_ led to an increased expression of oxidative-stress-related gene, heme oxygenase (decycling) 1 (HMOX_1_), which encodes HO in human bronchial epithelial cells. As aforementioned, an increased level of HO indicates increased OS. With exposure to NO_2_ and O_3_, the expression of HMOX_1_ and proinflammatory-related genes, including interleukin-6 and interleukin-8, increased [26]. More importantly, these molecules not only are deposited in the respiratory system but also travel to diverse organ systems through circulation [26–28].

OS and ROS could compromise male reproductive fucntion through altering the reproductive hormone profile and impairing spermatogenesis. ROS has been reported to be responsible for reduction in testosterone production, and a low testosterone level had a negative effect on spermatogenesis and male fertility [29–31]. Testosterone can protect the testicular microenvironment from damage by ROS [32, 33]. Activating hypothalamus-pituitary-gonadal (HPG) axis with leptin in rat could induce high FSH and LH levels. High levels of FSH and LH were associated with high OS and DNA fragementation [34]. Similar results were reported by Beattie et al. after administering exogenous LH to rat Leydig cells [35]. ROS, on the other hand, downregulates the HPG axis through hormonal cross-link through activation of the hypothalamus-pituitary-adrenal axis. Cortisone secretion increased in response to increased stress, a high level of which suppresses FSH and LH secretion, finally leading to decreased testosterone production [31].

Exposing an animal model to air pollution reduced its testosterone level [36]. However, in the present study, the toxic ambient pollutants did not show a statistically significant effect on the sex hormone profile in our study population. Calogero el al reported similar results. Compared with the control group, Tollgate workers exposed to a high level of car exhaust did not have significantly different serum levels of LH, FSH, and testosterone [37]. However, additional studies are required to evaluate OS by using markers, such as SOD and GPx, after exposure to ambient pollutants to further establish the link between ambient pollutants, OS, and male sexual endocrinological function.

Spermatozoon is a cell type that generates ROS independently, which is crucial in the acrosome reaction [38]. However, excessive ROS attacks the fluidity of the sperm plasma membrane and induces DNA damage in the sperm nucleus [27, 28]. Moreover, spermatozoa are more vulnerable to OS than oocytes because they lack downstream enzymes that participate in base excision repair [7, 11, 39, 40]. Human testicular development mainly occurs during puberty, and different cell types, including Sertoli cells, spermatogonia, and spermatocytes, play a role. In childhood, the elongation of seminiferous tubules contributes to testicular growth. In puberty, the testicles enlarge due to increase in the diameter of the seminiferous tubules [41, 42]. Later, the exponential growth of germ cells during spermatogenesis increases the testicular volume [21, 43].

The effect of OS on testicular development was studied in mice models. Mice exposed to excessive OS caused by crotonaldehyde, a food preservative, had significantly low levels of GPx and SOD with an increased malondialdehyde (MDA) level, which was a direct indicator of lipid peroxidation-induced injury caused by ROS. Following increased ROS and OS, lower testicular and epididymal weights were observed in the experimental group [44]. Furthermore, mice exposed to doxorubicin-induced OS had similar outcomes. After a week’s treatment, testicular volume, epididymal sperm count, and seminiferous tubule diameter decreased compared with the control group. Excessive OS and lipid peroxidation can lead to germ cell damage and testicular degeneration [45–47].

Among humans, the effect of OS on the seminal parameter and testicular volume were studied in patients with varicocele. Patients with varicocele had higer OS levels than healthy controls, and testicular biopsy showed that the semiferous tubule diameters of patients with varicocele were reduced [48, 49]. Hence, we adjusted the multivariate linear regression models with the presence of varicocele.

Our study could not evaluate the OS alteration in the male reproductive system that results from ambient pollutant exposure. A future study with OS markers from the semen testicular tissue after exposure to ambient pollution should be conducted. If semen quality and testicular volume change, the sperm structure should be observed in relation to increased OS to further establish the link between OS and the male reproductive system. In participants exposed to ambient pollutants, the enzymes that partake in redox could be analyzed, including SOD, GPx, MDA, and HO. Furthermore, DNA fragmentation could be analyzed, as it has been reported to result from OS and could lead to sperm apoptosis [27, 28, 38, 50].

To our knowledge, this is the first study to evaluate the effects of ambient pollutants on the testicular volume of men with infertility. Nevertheless, the current study has several limitations. First, we considered the exposure variation based on daily average exposure concentrations for a specific period (2 years) while ignoring the effects of the peak and exposure of certain pollutants within a day. Additionally, we estimated individual exposure based on the residential address record at the clinic. This ignored occupational exposure, which could be a serious confounding factor when occupational exposure to air pollution is profound; for example, if a participant is a factory worker. Moreover, if the patient spends only a little time at home, estimating the exposure based on residential address could be misleading. Second, our study was retrospective and observatory in design because intentionally exposing participants to air pollutants is unethical. Moreover, the present study lacked serum or seminal marker for OS, which we believe participates in the alteration of seminal parameter and testicular volume. We aim to conduct further studies by using surrogate markers to determine OS in the semen and testicular tissue of patients with infertility who needed testicle biopsy. Third, our study population was relatively small, and some data were missing. Therefore, the statistical power to detect the effects of pollutants on the reproductive system and sex hormone profile may be weakened. Selection bias was inevitable, as most of our patients were from northern Taiwan. Moreover, as data were obtained from men with infertility, a future study with a healthy population is needed to extrapolate the result to the general population. Recruiting patients from other parts of Taiwan and data from healthy individuals, such as from volunteers undergoing health examination during college entrance, are recommended to strengthen the statistical power.

## Conclusions

The result of the present study suggests that ambient NO_x_ are a risk factor for poor sperm motility and decreased sperm concentration. Moreover, exposure to SO_2_ was negatively associated with testicular volume. However, the strength of the result is limited by the retrospective nature and small sample size of the study. Future prospective studies with healthy control and oxidative markers should be conducted to further establish the cause–effect relation between ambient pollutants and the male reproductive system and determine the mechanism behind it.

## Data Availability

The datasets used in the current study can be obtained from the corresponding author on reasonable request.

## Abbreviations

WHO: World Health Organization
Ppb: Parts per billion
PM_2.5_: Particulate matters 2.5
O_3_: Ozone
SO_2_: Sulfur dioxide
NO: Nitrogen monoxide
NO_2_: Nitrogen dioxide
FSH: Follicle stimulating hormone
LH: Luteinizing hormone
BMI: Body mass index
SOD: Superoxide dismutase
HO: Heme oxygenase
ROS: Reactive oxygen species
HMOX_1_: Heme oxygenase (decycling) 1
GPx: Glutathione peroxidase
MDA: Malondialdehyde
NOx: Nitrogen oxides
OS: Oxidative stress
HPG: Hypothalamus-pituitary-gonadal
